# Electrophysiological Alterations in the Progression of Parkinson's Disease and the Therapeutic Effect of Tetrabenazine on Rats With Levodopa‐Induced Dyskinesia

**DOI:** 10.1111/cns.70250

**Published:** 2025-02-06

**Authors:** Yuewei Bi, Pengfei Wang, Min Li, Zhuyong Wang, Siyuan Lv, Yong Yang, Wangming Zhang

**Affiliations:** ^1^ Department of Neurosurgery Peking Union Medical College Hospital, Chinese Academy of Medical Sciences and Peking Union Medical College Beijing P. R. China; ^2^ Neurosurgery Center, Department of Pediatric Neurosurgery, Guangdong Provincial Key Laboratory on Brain Function Repair and Regeneration, Zhujiang Hospital Southern Medical University Guangzhou P. R. China; ^3^ Department of Otology The First Affiliated Hospital of Zhengzhou University Zhengzhou P. R. China

**Keywords:** functional connectivity, gamma oscillation, Granger causality, levodopa‐induced dyskinesia, Parkinson's disease, tetrabenazine

## Abstract

**Aims:**

Dopamine replacement therapy is the backbone of Parkinson's disease (PD) treatment. However, long‐term levodopa (l‐DOPA) administration can lead to the severely disabling motor complication L‐DOPA‐induced dyskinesia (LID), for which standard, effective therapy is currently lacking. This study was conducted to characterize the distinct neural electrophysiological patterns involved in the progression of PD and to examine the efficacy of tetrabenazine, a vesicular monoamine transporter‐2 inhibitor, in alleviating dyskinesia and its underlying electrophysiological mechanism.

**Methods:**

Electrophysiological analysis was performed to obtain power spectrum density and functional connectivity information from local field potential (LFP) data recorded from the primary motor cortex (M1) and dorsolateral striatum (DLS) during different pathological states in PD model rats. Behavioral tests and abnormal involuntary movements (AIMs) scoring were conducted to confirm PD model establishment and assess LID severity.

**Results:**

Increased beta oscillations and abnormally strengthened beta causality in the M1 → DLS direction and exaggerated beta‐band M1–DLS functional connectivity were observed in the PD state. l‐DOPA administration suppressed beta activity and augmented gamma power in the M1 and DLS, with increased gamma causality in the M1 → DLS direction and beta causality in the DLS → M1 direction, as well as elevated gamma‐band M1–DLS functional connectivity. Tetrabenazine strongly ameliorated dyskinetic manifestations. It suppressed gamma power in the M1 and DLS, reduced gamma causality and increased beta causality in the M1 → DLS direction, reduced beta causality in the DLS → M1 direction, and reduced gamma‐band M1–DLS functional connectivity.

**Conclusion:**

Tetrabenazine abrogated aberrant gamma activity to improve LID symptoms, which provides compelling evidence for its future clinical application in LID therapy.

## Graphical abstract

The distinct neural electrophysiological patterns in the PD and LID states. The abnormal functional connectivity and information flow direction in PD state. The cumulative effects of chronic l‐dopa administration on functional connectivity and information flow direction in LID states. The potential mechanism of tetrabenazine's amelioration of dyskinesia at the electrophysiological level in rats.

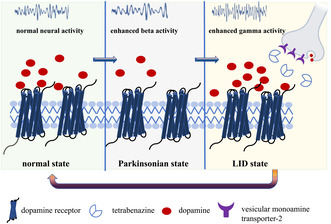

## 1 Introduction

Parkinson's disease (PD) is a neurodegenerative disorder commonly observed in older adults and affects approximately 1% of the population over the age of 60. However, rare cases can also occur in individuals under 50 and even under 40. Although the prevalence of PD increases significantly with age, the occurrence of early‐onset PD highlights the diversity of the condition and its complex pathological mechanisms [1]. It is characterized by an imbalance in neural activity in the cortico‐basal ganglia‐thalamo‐cortical (CBTC) circuit, which plays key roles in the regulation of cognitive and motor functions [2]. In clinical practice, dopamine replacement therapy (DRT) is the most effective option for the management of PD [3]. However, long‐term levodopa (L‐DOPA) administration can reduce the clinical efficacy of the drug and lead to the occurrence of a series of adverse secondary effects [4]. L‐DOPA‐induced dyskinesia (LID) is a major, severely disabling, motor complication of chronic L‐DOPA use [5]. It has severe impacts on patients' quality of life and imposes major burdens on their family members and society as a whole [6]. The disequilibrium of neural network excitatory and inhibitory signaling underlies the occurrence of LID, but the pathophysiological mechanism of this disease remains obscure, and standard, effective therapeutic regimens are lacking.

Pathologically increased beta oscillation (at 13–35 Hz) in the CBTC circuit is currently regarded as a crucial electrophysiological biomarker of PD [7]. This increased oscillation inhibits motor function and is related to the severity of akinetic symptoms, possibly due to pathological neural network formation in the complex subthalamic nucleus (STN)–cortical loop and the effects of beta bursts on the pathophysiological process of PD [8, 9, 10]. DRT can disturb these abnormal neural activities to improve parkinsonian manifestations. In contrast, gamma oscillation is considered prokinetic, and greater gamma oscillation is correlated with motor improvement in patients with PD [11]. Güttler et al. reported LID responses to the sustained increase in gamma oscillation in the primary motor cortex (M1) in patients with PD treated with l‐DOPA [12]. Neurophysiological activity in patients with LID is characterized by a reduction in beta‐band power and overactive gamma oscillation (at 60–90 Hz), which disrupts motor loop function [13, 14]. This excessive increase in gamma oscillation related to dyskinesia can be used as a control signal for adaptive deep brain stimulation (DBS) [15].

Functional connectivity can capture the characteristics of distribution differences and quantitatively describe the correlation of time‐dependent activity between spatially distant neuronal units, which provides a theoretical basis for neural network–based research [16]. It has expanded our understanding of the brain regions and neural circuits involved in PD manifestations and complications [17]. Functional connectivity has emerged as a valuable predictor of clinical outcomes, independently providing insights into the effectiveness of treatments. It can be utilized to identify suitable candidates for DBS among individuals with early‐stage PD [18, 19] while also offering predictive value regarding the potential therapeutic benefits of such interventions [20, 21, 22]. This approach underscores the importance of personalized treatment strategies in managing the disease. Functional brain network alterations are also related to the severity of motor and cognitive symptoms in subjects with PD [23, 24, 25]. Moreover, by evaluating the impact of dopamine on the connectivity between the primary sensorimotor cortex and the putamen, researchers can predict both the likelihood and severity of dyskinesia in PD patients. Additionally, an unusually strong connection between the dentate nucleus of the cerebellum and the putamen may play a significant role in the pathophysiology of peak‐dose dyskinesia. This understanding provides further insight into the mechanisms underlying motor complications in PD, potentially guiding more targeted therapeutic approaches [25]. Although previous studies have revealed abnormal neural oscillatory flow in LID states, the interactions among large‐scale brain networks in the context of LID has not been clarified. The exploration of dynamic neural network connectivity could provide evidence for abnormalities in brain functional integration and specialization in LID. In addition, a precise understanding of gamma‐band functional connectivity would support further investigation of the pathophysiology of LID and the prediction of clinical outcomes.

Granger causality analysis can be used to illustrate causal relationships among brain regions and to assess connectivity and input–output interactions among neural networks [26]. Moreover, it offers a robust approach for inferring causal relationships between time‐series variables through statistical hypothesis testing. This method not only quantifies the strength and significance of these causal connections but can also be seamlessly integrated into various statistical models, such as multivariate regression frameworks. Its versatility allows it to be applied across different data types and domains. By identifying key causal variables, Granger causality analysis increases the predictive accuracy of forecasting models and provides valuable insights into the temporal dynamics of complex systems [27, 28, 29]. PD is characterized by increased activity but reduced information flow in the cortical network [30], and a recent study revealed that information flow and functional interactions in deep white matter networks are negatively associated with PD motor and nonmotor symptoms [31]. A Granger causality analysis revealed that activity in the STN → M1 direction increased during high‐conflict trials in subjects with PD [32]. Our team demonstrated that long‐term l‐DOPA use enhanced theta synchronization and increased information flow in the substantia nigra pars reticulata (SNr) → M1 direction [33]. However, gamma‐band information flow in the CBTC circuit in the dyskinetic state and the cumulative effect of chronic l‐DOPA use on pathological neural network interactions remain obscure.

Tetrabenazine, approved by the US Food and Drug Administration for the treatment of hyperkinetic movement disorder Huntington's disease (HD), is used primarily to manage HD–induced chorea [34], likely by preventing dopamine from binding to upregulated D2 receptors; however, it has been suggested that tetrabenazine can inhibit vesicular monoamine transporter‐2 (VMAT‐2) [35]. VMATs are vital for monoamine (including dopamine)‐mediated synaptic transport from the cytoplasm into synaptic vesicles and enable monoamine accumulation, storage, and eventual release from synaptic vesicles via exocytosis, whereas VMAT‐2 inhibitors deplete monoamines in presynaptic vesicles and prevent their release [36]. Like HD, LID involves exaggerated phasic dopamine signaling in the basal ganglia [37]. It has been proven that tetrabenazine has a therapeutic effect on dyskinetic advanced PD patients, as reflected by changes in the dyskinesia score of the Unified Parkinson's Disease Rating Scale (UPDRS) [38]. The latest research shows that tetrabenazine reduces abnormal striatal dopamine transfer and the aberrant release of exogenous l‐DOPA from neural synapses, supporting its clinical potential to counteract LID development [39]. However, the underlying electrophysiological neural mechanism by which tetrabenazine can ameliorate dyskinesia symptoms in the LID state remains unknown.

In this study, we characterized the distinct neural electrophysiological patterns that occur in the PD and LID states and assessed the efficacy of tetrabenazine in alleviating hyperkinesia in rats with LID. We also explored the mechanism underlying the effects of tetrabenazine at the electrophysiological level for the first time.

## 2 Materials and Methods

### 2.1 Animals

All experimental procedures performed as part of this research adhered to the National Institutes of Health Guidelines for the Care and Use of Laboratory Animals (Publication No. 8023, revised 1978). The study was conducted with 65 healthy male Sprague–Dawley rats aged 8–10 weeks and weighing 280–300 g, purchased from the Experimental Animal Center of the Southern Medical University of China. The rats were housed under a standard 12/12‐h light/dark cycle (lights on at 8:00 a.m.) and fed separately after microelectrode implantation, as applicable. To maintain a stable body weight, food intake was limited to 10 g/day. Appropriate measures were taken to relieve the discomfort of the rats.

### 2.2 Behavioral Tests

All the rats were transferred to the testing room 1 h before the test to allow them to adapt to the testing environment, reducing stress caused by the new environment. Before test initiation, the rats were placed in cages for 5 min for habituation. Measures were adopted to ensure that the experimental room was quiet to avoid noise disturbances. For all the animal experiments, a digital camera was installed above the testing area to record the rats' movements and activities. Before and after the experiment, the test chamber was wiped with 75% alcohol to ensure that it was clean and odorless. The rats were observed by an observer blinded to the experimental groups and interventions, and repeated measures of the same data obtained by the observer at different points in time (data not shown) were performed to test the reliability of the observer and confirm the robustness and reproducibility of the data analysis process.

For the open‐field test (OFT), a square testing apparatus (100 cm × 100 cm), made of transparent plastic, with black sidewalls 40 cm in height, was prepared. A camera was installed above the testing area to record the rats' behavioral trajectories and activities. A rat was gently grasped at the base of its tail, approximately 1/3 from the end, and lifted out of its cage. The rat faced away from the experimental personnel to avoid causing stress through rough handling. The rat was quickly and gently placed in the center of the test chamber, and the video capture and analysis system was simultaneously started to automatically record the locomotor activity of the rat in the open field. The observer left the behavioral testing room and entered another area where the rat could still be observed. Each rat was allowed to move freely for 5 min, and the total distance traveled and velocity were measured using Noldus EthoVision XT software (Noldus, the Netherlands) to assess locomotor activity. Then, the video recording was stopped after the experiment. The rat was removed from the test chamber and returned to its cage.

In the cylinder test, each rat was gently placed in an open transparent plastic cylinder facing the wall. A camera was installed above the testing area to record the behavior of the rats. The animal's behavior, particularly the use of its forelimbs, was recorded as it freely explored the cylinder. We observed whether the animal could freely turn, stand, and climb the walls of the cylinder. The number of times the rat used both forelimbs symmetrically when standing to support its weight, as well as when grasping or pushing, was recorded. Twenty forelimb contacts, which were defined as the placement of the entire forepaw against the cylinder wall, indicating body support, were recorded. The percentage of contacts of the forelimb contralateral to the lesioned side (left) of all 20 contacts was calculated.

In the apomorphine (APO) challenge experiment, the specific number of rotations performed by each rat over a 5‐min interval, commencing precisely 15 min postinjection of APO, was meticulously recorded and analyzed. This assessment served as a quantitative measure of the behavioral response to the drug, reflecting the functional status of the dopaminergic system in the lesioned hemisphere. The criterion for establishing a sufficient lesion was stringently defined as the exhibition of more than 30 rotations toward the contralateral (nonlesioned) side during this period.

The evaluation of l‐DOPA‐induced abnormal involuntary movements (AIMs) was conducted with a standardized AIM scoring system to quantify the extent and nature of the observed dyskinesia. This comprehensive assessment encompassed the severity of motor, forelimb, and orolingual dyskinesia, with each category being graded on a well‐defined scale ranging from 0 to 4. A score of 0 represented the absence of any abnormal movement, whereas progressively higher scores indicated increasing severity and persistence of dyskinesia. Specifically, a score of 1 denoted dyskinesia occurring during less than 50% of the observation period, whereas a score of 2 signified dyskinesia present for at least half of the session. Scores of 3 and 4, respectively, reflected continuous dyskinesia that could be temporarily interrupted by external means and constant, uninterruptible dyskinesia. This scoring was conducted at 2‐min intervals, captured within 10 separate 20‐min video‐recorded sessions, amounting to a total potential observation time of 180 min following l‐DOPA injection. The cumulative maximum score achievable across all sessions was 120, providing a comprehensive overview of the dyskinetic manifestations over time. Additionally, response times (RTs), defined as the elapsed intervals between the administration of l‐DOPA and the initial manifestation of dyskinetic symptoms, were meticulously recorded. This recording process adhered to previously established protocols, ensuring consistency and reliability in the measurement of the onset of AIMs [40].

### 2.3 Experimental Procedures

#### 2.3.1 Experiment 1

Experiment 1, as depicted in Figure S1A, was conducted with a group of 25 rats. On Day 1, a unilateral PD model was induced by administering 6‐hydroxydopamine (6‐OHDA; 4 μg/μL, 5 μL in saline; DR0610; HarveyBio, Beijing, China) into the right medial forebrain bundle (MFB). To assess the success of the PD model, behavioral evaluations were performed on Days 12, 13, and 14 using the OFT, cylinder test, and APO (0.75 mg/kg, intraperitoneal [i.p.]; Macklin, Shanghai, China) challenge, respectively. Successful modeling was confirmed in 24 of the rats, which were then subjected to a 14‐day treatment regimen with ‐DOPA (8 mg/kg, i.p.; Macklin) and benserazide (12 mg/kg, i.p.; Macklin) (referred to as LB). On Day 29, these rats were randomly assigned to one of four groups, each receiving a different dose of tetrabenazine (0, 2, 4, or 6 mg/kg; T135203, Aladdin, Shanghai, China) alongside continued LB treatment. The objective was to determine the optimal tetrabenazine dose for further experiments. Throughout the study, video recordings and assessments using the AIM scale were performed on Days 15, 21, 28, and 29. After the experimental procedures were complete, the rats were euthanized, and their brain tissue was collected for histological analysis.

#### 2.3.2 Experiment 2

In Experiment 2, depicted in Figure S1A, a total of 40 rats were used and randomly assigned to either the PD group (*n* = 30) or the sham group (*n* = 10). On Day 1, 6‐OHDA (4 μg/μL, 5 μL in 0.9% w/v saline; HarveyBio) was injected into the right MFB to induce unilateral PD, whereas the sham group received a saline injection (5 μL; Jiangxi Kelun Pharmaceutical Co. Ltd., Fuzhou, China) into the same area. To evaluate the efficacy of the PD model, assessments were carried out on Days 12, 13, and 14 using the OFT, cylinder test, and APO (APO; 0.75 mg/kg, i.p.; Macklin) challenge. Successful modeling was confirmed in 25 out of 30 PD group rats.

On Day 15, microelectrodes were implanted in both the PD and sham group rats, followed by a 2‐week recovery period. By Day 29, a standardized lesioning procedure was performed on all PD model groups. The PD group rats were then randomly assigned to one of two treatment groups: the PD + saline group (*n* = 8), which received saline (2.4 mL, i.p.), or the PD + LB group (*n* = 17), which was treated with l‐DOPA (8 mg/kg, dissolved in 0.9% w/v saline, i.p.; Macklin) and benserazide (12 mg/kg, dissolved in 0.9% w/v saline, i.p.; Macklin) for 14 consecutive days.

On Day 43, a stable LID model was established in the PD + LB group, which was subsequently divided into two subgroups: the LID + LB group (*n* = 9), receiving LB alone, and the LID + T group (*n* = 8), receiving LB in combination with tetrabenazine (4 mg/kg, dissolved in 0.9% w/v saline, i.p.; Macklin) for 7 days. Throughout the study, AIM scoring and video recordings were performed on Days 29, 32, 35, 38, 41, 43, 45, and 49. Additionally, local field potential (LFP) recordings were conducted on Days 41 and 49. The OFT was performed on Days 42 and 48, 80 min after the injection of LB or LB plus tetrabenazine.

After all electrophysiological recordings were completed, the rats were euthanized, and their brain tissues were harvested for histological examination.

### 2.4 Surgeries

For the induction of unilateral lesions and the implantation of microelectrodes, the rats were first anesthetized with sodium pentobarbital (50 mg/kg, dissolved in 0.9% w/v saline, i.p.; Sigma, St. Louis, MO, USA). The head of each rat was then secured in a stereotaxic frame. Stereotaxic coordinates were determined on the basis of the atlas by Paxinos and Watson (2007), specifically: the MFB coordinates were –1.8 mm anteroposterior (AP), −2.0 mm mediolateral (ML), and − 8.35 mm dorsoventral (DV; below the skull surface); the right M1 Layers V and VI coordinates were +2.6 mm AP, −2.6 mm ML, and −1.5 to 1.6 mm DV (below the dura); and the right dorsolateral striatum (DLS) coordinates were 0 mm AP, −3.5 mm ML, and −5.5 mm DV (below the dura).

For the implantation of the microelectrodes, two arrays, each containing eight stainless‐steel microwires insulated with Teflon (50 μm diameter, arranged in a 2 × 4 configuration with 150 μm spacing between wires; Plexon, Hong Kong Special Administrative Region, China), were inserted vertically into the right M1 and DLS. Grounding was achieved by wrapping wires around two screws placed above the cerebellum, ensuring contact with the dura mater. The electrodes were then fixed in place with screws and dental cement.

To prevent postsurgical infections, all the rats received daily injections of penicillin (8000 units/mL, 1 mL, i.p., dissolved in normal saline; China Animal Husbandry Industry Co. Ltd., Beijing, China) for a period of 7 days following electrode implantation.

### 2.5 Electrophysiological Recording

Data from the M1 and DLS regions were captured using a 128‐channel data acquisition system (Plexon). Baseline LFPs were recorded 5 min prior to LB injections on Day 29 (D0) to establish the parkinsonian baseline. Subsequent LFP recordings were taken at eleven 5‐min intervals—one before and 10 after LB administration—at 20‐min intervals over a 180‐min period—on Days 41 (D13) and 49 (D21). The recordings were performed at a sampling rate of 1 kHz, with an amplification factor of 300×, and were bandpass filtered between 0.5 and 1000 Hz. A ground wire served as the reference electrode throughout the recording sessions.

### 2.6 Histological Analysis

Following anesthesia with sodium pentobarbital (50 mg/kg, i.p.; Sigma), a 20‐μA current was applied through the recording electrodes for 30 s to mark the sites of microelectrode implantation. The rats were subsequently perfused transcardially with 500 mL of precooled 0.9% saline, followed by 500 mL of ice‐cold 4% paraformaldehyde. After perfusion, the brains were extracted and fixed in 4% paraformaldehyde for 24 h. The brains were then dehydrated and embedded in paraffin.

Coronal sections (8 μm thick), including sections from the M1, DLS, and substantia nigra pars compacta (SNc), were prepared using a paraffin microtome (Leica, Germany). To visualize the electrode tracts, sections from the M1 and DLS were mounted on glass slides, stained with hematoxylin and eosin (HE), and examined with a digital slide scanner (PANNORAMIC; 3DHISTECH Ltd.). Animals with mistargeted electrodes that were implanted outside the target brain area M1 and DLS, as determined by tissue sectioning, were excluded. No animals were excluded on the basis of this criterion in this study.

### 2.7 Immunofluorescence Staining

To assess the extent of lesions in the SNc, immunofluorescence staining for tyrosine hydroxylase (TH) was conducted. First, the sections were placed in a drying oven at 65°C for 90 min, followed by three 10‐min rinses in dimethylbenzene for paraffin removal. The tissue was then rehydrated through a series of ethanol solutions (100%, 95%, 85%, and 75% ethanol, each for 5 min) and double‐distilled water (5 min, repeated three times).

Antigen retrieval was performed by submerging the sections in citrate solution (Beyotime, Shanghai, China) and heating them in a microwave until boiling, after which they were boiled for 15 min. After cooling to room temperature, the sections were washed three times with phosphate‐buffered saline (PBS; 5 min each). Next, the sections were incubated in 5% normal donkey serum (017‐000‐121‐E; Jackson, Lancaster, PA, USA) diluted in PBS with 0.03% Triton X‐100 for 1 h at room temperature. The samples were then incubated overnight at 4°C in a solution containing rabbit anti‐TH polyclonal primary antibody (1:500; Abcam, Cambridge, UK, Cat# EP1532Y, RRID:AB_2801410). The following day, the sections were incubated at room temperature for 2 h with an Alexa Fluor 594‐conjugated donkey anti‐rabbit IgG secondary antibody (1:500; 711‐585‐152; Jackson) and then washed three times with PBS. After a final set of rinses (5 min each), the sections were stained with DAPI (C0065‐10; Solarbio, Beijing, China) for 3 min at room temperature. Images were captured using an ECLIPSE Ti2‐E inverted microscope (Nikon Corporation, Tokyo, Japan) and analyzed with ImageJ software (National Institutes of Health, USA) to evaluate the TH labeling intensity and the number of TH+ cells.

### 2.8 Data Analysis

All data analysis was conducted via MATLAB (R2021a; MathWorks, Natick, MA, USA).

#### 2.8.1 Preprocessing

The data were preprocessed via the Fieldtrip toolbox [41]. Data from the 5‐min LFP sessions were divided into 300 1‐s segments using the ft_definetrial function. Then, the artifacts were identified and removed using the ft_artifact_zvalue and ft_rejectartifact functions, respectively.

#### 2.8.2 Spectral Analysis of LFPs

Baseline LFPs recorded 5 min prior to l‐DOPA administration on Day 29 were analyzed to estimate power spectrum densities (PSDs) for both the sham and PD groups. On Day 41, LFPs collected 80 min after lb administration, corresponding to peak dyskinesia, were used to calculate PSDs for the PD + saline and PD + LB groups. The PSDs were computed using the ft_freqanalysis function from the Fieldtrip toolbox, which uses fast Fourier transform.

For this analysis, PSDs were derived from one hundred and twenty 1‐s segments of processed data. The settings included a frequency range of 1–150 Hz (cfg.foilim = 1:1:150), a spectral smoothing parameter (tapsmofrq) of 2, and a Hanning window for tapering.

#### 2.8.3 Assessment of Functional Connectivity

To assess functional connectivity between the M1 and the DLS, we utilized the ft_connectivityanalysis function in Fieldtrip. We performed three types of analyses: coherence, the debiased weighted phase lag index (dwPLI), and Granger causality. The following calculation parameters were used for these analyses: cfg.foilim = 1:150 and cfg.tapsmofrq = 5.

The functional connections between the M1 and the DLS were assessed using coherence analysis. Coherence is a standardized metric that quantifies the linear correlation between signals in the frequency domain, with values ranging from 0 (indicating no linear relationship) to 1 (indicating a perfect linear relationship).

We applied Granger causality analysis to explore the direction of information flow across different rhythmic activities, shedding light on the causal interactions between the M1 and the DLS. Specifically, Granger causality was estimated in the frequency domain, a widely used approach to determine whether one time series can predict another. In simple terms, variable X is said to Granger‐cause variable Y if the past values of X improve the prediction of Y's future beyond what can be predicted using only Y's past values. This method provides a statistical framework for analyzing directed functional connectivity based on observed data [33].

The dwPLI assessed frequency‐based spatial phase synchronization, allowing for a more precise analysis of phase relationships without being biased by volume conduction effects [42].

### 2.9 Statistical Analysis

All the data are reported as the means ± standard errors of the means. All the data were tested for a normal distribution before statistical analysis. Unpaired *t*‐tests or Kolmogorov–Smirnov tests were used to compare the behavioral test and immunofluorescence results, AIM scores, RTs, PSDs, Granger causality, coherence, and dwPLI between the rats in the sham and PD groups, the PD + saline and PD + LB groups, the LID + LB and LID + T groups, and the LID + E and LID + T groups, depending on whether the data conformed to a normal distribution or not. One‐way analysis of variance (ANOVA) with Tukey's multiple comparison test was used to compare the effects of different concentrations of tetrabenazine. For PSDs, Granger causality, coherence, dwPLI, and areas under the curve for theta‐, beta‐, and gamma‐band oscillations were calculated. For PSDs, normalization was performed on the LFP data collected on Days 43 and 49 by calculating the rates of the areas under the curve in the theta‐, beta‐, and gamma‐band oscillations on Days 43, 29, 49 and 43, respectively. The total motor distance and velocity of LID + T group rats on Day 14 (PD state, PD), Day 42 (LID state, PD + LB), and Day 48 (post‐tetrabenazine intervention state, LID + T) in the OFT were subjected to repeated‐measures one‐way ANOVA, and Tukey's multiple comparison test was used to reveal significant differences between groups. *p* values < 0.05 were considered to indicate statistical significance. GraphPad Prism 9.0 software (Version 9.0.0 for Windows; GraphPad Software, Boston, MA, USA; www.graphpad.com) was used to conduct the analyses.

### 2.10 Ethics Statement

All experimental procedures performed were approved by the Institutional Animal Ethics Committee of the Southern Medical University of China (LAEC‐2022‐146).

## 3 Results

### 3.1 PD Model Establishment

**FIGURE 1:**
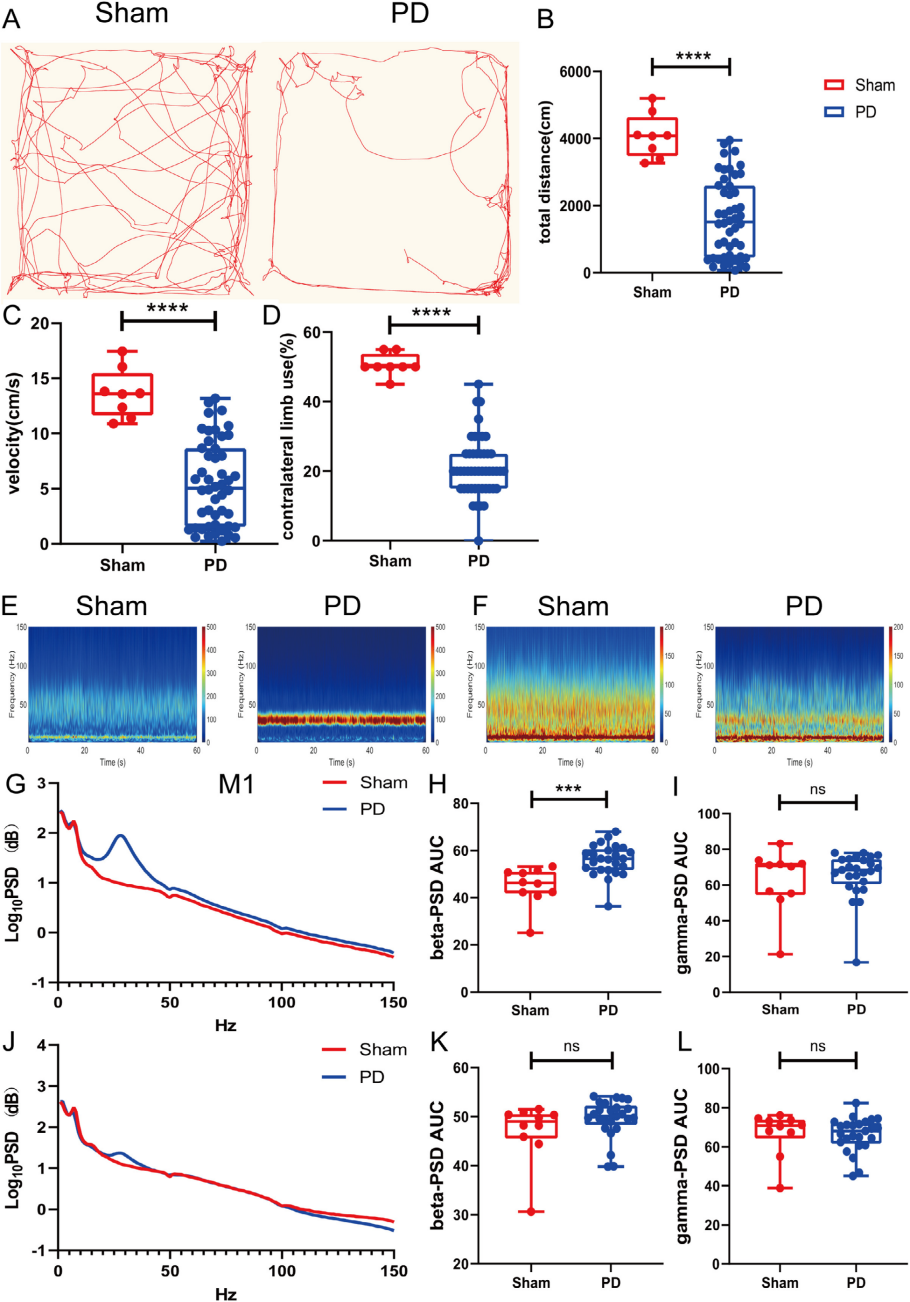
M1–DLS oscillation in the PD state. (A, B and C) Representative motion trails, total distance, and velocity traveled in the OFT. (D) Contralateral limb use in the cylinder test. (E and F) Representative M1 and DLS PSD spectrum plots. (G and J) PSDs of LFPs in the M1 and DLS. (H, I, K, and L) Summaries of beta‐ and gamma‐band oscillation in the M1 and DLS. Data are means ± SEMs. ***p < 0.001, **** *p* < 0.0001, Kolmogorov–Smirnov test and unpaired *t*‐tests (sham, *N* = 10; PD, *N* = 25). DLS, dorsolateral striatum; LFP, local field potential; M1, motor cortex; OFT, open field test; PSD, power spectrum densities; SEM, standard errors of mean.

The APO challenge indicated that PD lesions were successfully created in 49 of 55 rats. The rats with PD exhibited reduced movement distance and velocity in the OFT, which was consistent with parkinsonian symptoms (both *p* < 0.0001; Figure 1A–C). In the cylinder test, they avoided contralateral limb use (*p* < 0.0001; Figure S1D). Rats with 6‐OHDA‐induced lesions exhibited prominent dopaminergic neuron degeneration in the SNc, as indicated by decreased TH fluorescence intensity and TH^+^ cell numbers (both *p* < 0.0001; Figure S1C–E). HE staining confirmed that all electrodes were implanted at the appropriate positions (Figure S1F,G).

### 3.2 Exaggerated Beta Oscillation in the M1 in the PD State

The beta power in the M1 was greater in the PD group than in the sham group (*p* = 0.0002; Figure 1E,G,H). The gamma oscillation power did not differ between the groups (*p* = 0.63; Figure 1I). The beta and gamma powers in the DLS did not differ between the sham and PD groups (beta, *p* = 0.4574; gamma, *p* = 0.63; Figure 1F,J–L).

### 3.3 Aberrant Functional Connectivity in the CBTC Circuit in the PD State

**FIGURE 2:**
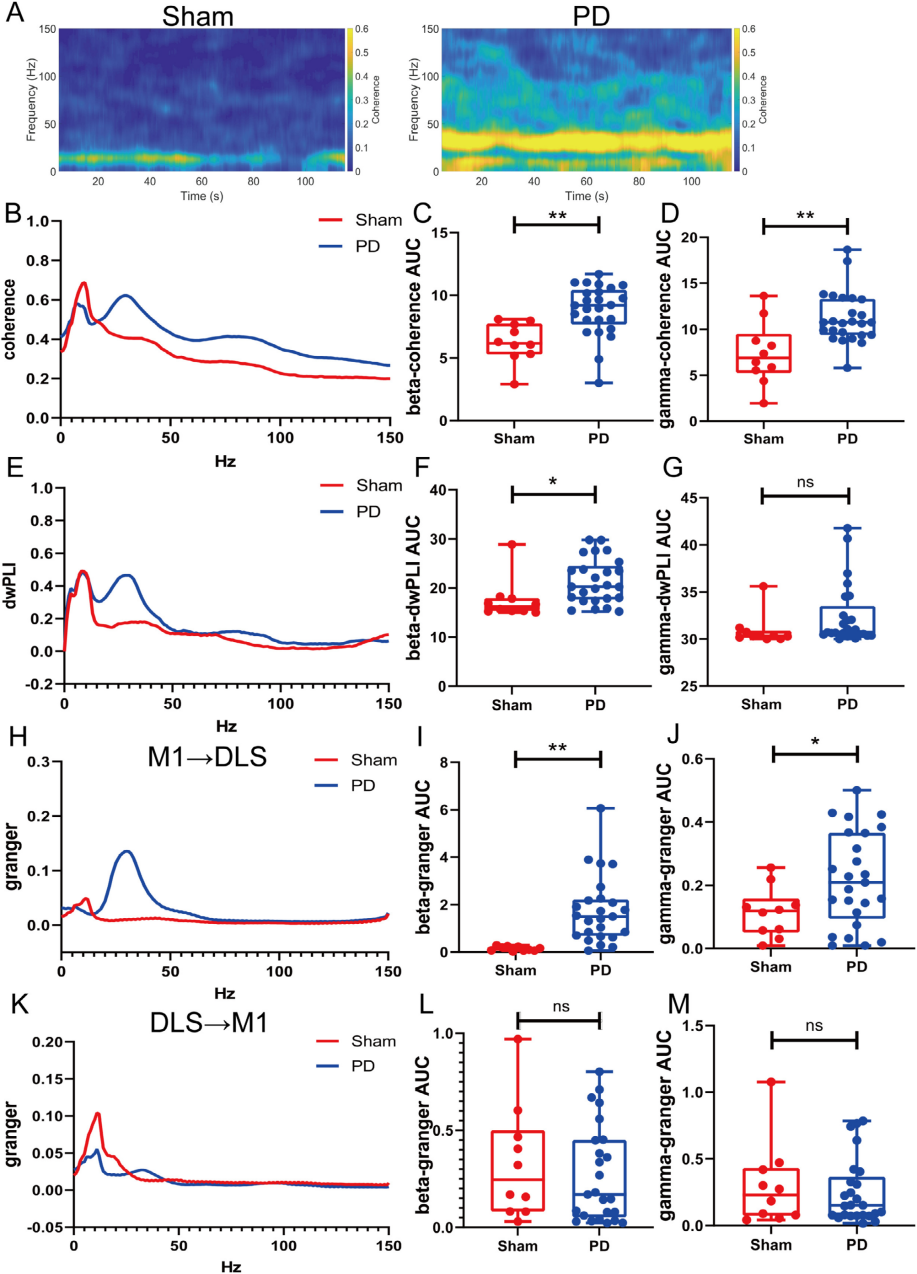
Functional connectivity in the CBTC circuit in the PD state. (A) Representative spectrum plots of coherence between the M1 and DLS. (B) M1–DLS coherence. (C and D) Summaries of coherence in the beta and gamma bands. (E) M1–DLS dwPLIs. (F and G) Summaries of beta‐ and gamma‐band dwPLIs. (H–J) M1 → DLS causality. (K–M) DLS → M1 causality. Data are means ± SEMs. *p < 0.05 and **p < 0.01, Kolmogorov–Smirnov and unpaired *t*‐tests (sham, *N* = 10; PD, *N* = 25). DLS, dorsolateral striatum; M1, motor cortex; PD, Parkinson's disease; SEM, standard errors of mean.

Beta‐ and gamma‐band coherence was stronger in the PD group than in the sham group (beta: *p* = 0.0012; gamma: *p* = 0.0012; Figure 2A–D). The beta dwPLI was greater in the PD group (*p* = 0.0227; Figure 2E,F), whereas no difference was observed for the gamma band (*p* = 0.0562; Figure 2G). Beta and gamma causalities in the M1 → DLS direction were significantly greater in the PD group than in the sham group (beta, *p* = 0.0012; gamma, *p* = 0.0422; Figure 2H–J). No difference was observed between groups in beta or gamma oscillations in the DLS → M1 direction (beta, *p* = 0.7197; gamma, *p* = 0.9748; Figure 2K–M).

### 3.4 Exaggerated Gamma Oscillation in the M1 and DLS Induced by l‐DOPA

**FIGURE 3:**
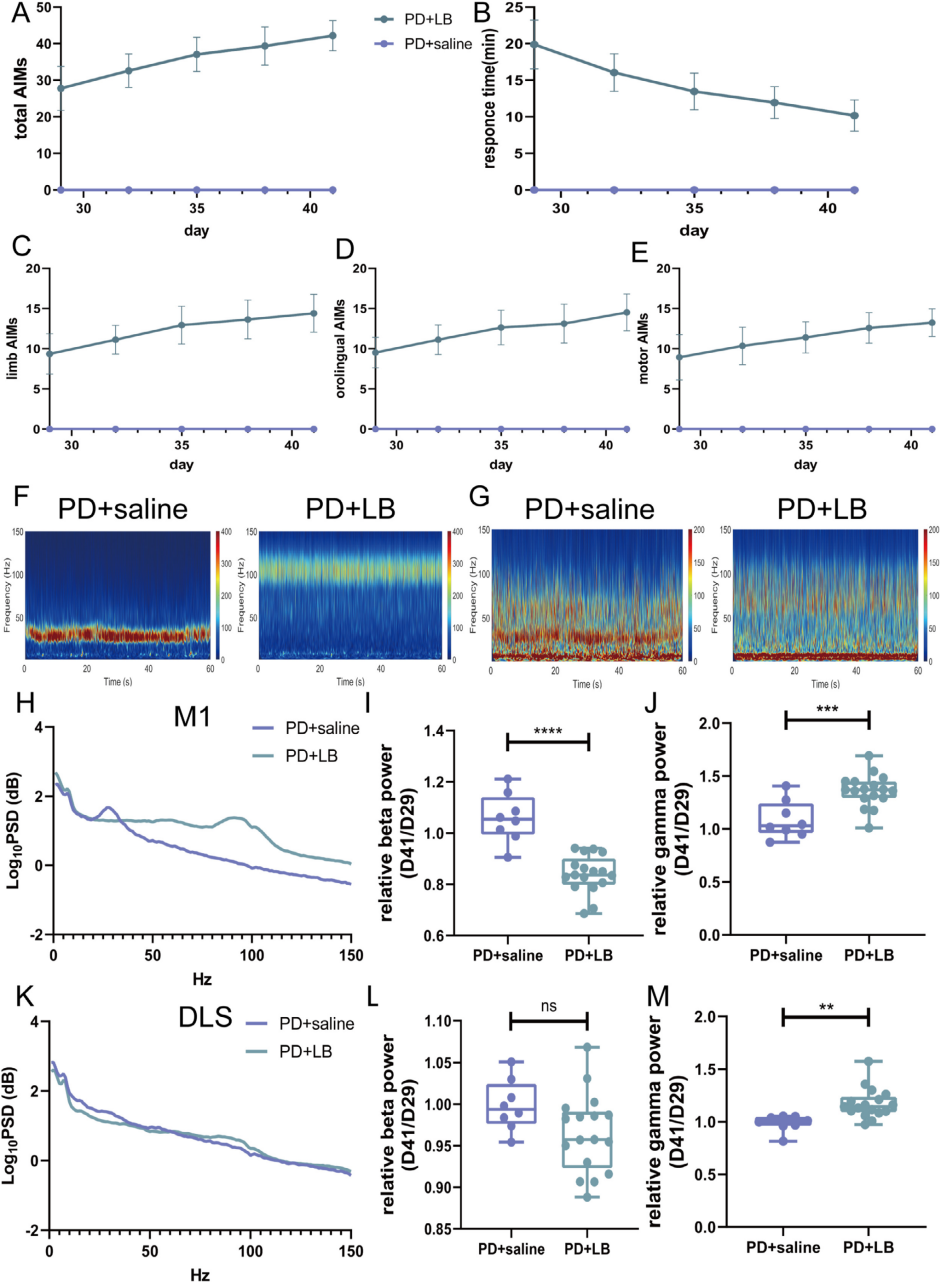
Oscillations in the M1 and DLS in the LID state. (A) Total AIMS scores after LB priming. (B) RTs after LB administration. (C–E) AIMS subscale scores after LB priming in the PD + saline and PD + LB groups. (F and G) Representative M1 and DLS PSD spectrum plots from the PD + saline and PD + LB groups. (H and K) PSDs of LFPs in the M1 and DLS. (I, J, L, and M) Summaries of normalized beta‐ and gamma‐band oscillation power in the M1 and DLS. Data are means ± SEMs. **p < 0.01, ***p < 0.001, and ****p < 0001, unpaired *t*‐test (PD + saline, *N* = 8; PD + LB, *N* = 17). AIMs, Abnormal Involuntary Movement Scale; DLS, dorsolateral striatum; LB, levodopa plus benserazide; LFP, local field potential; M1, motor cortex; PD, Parkinson's disease; PSD, power spectrum densities; RTs, response times; SEM, standard errors of mean.

The AIM scores increased as the disease progressed, which was accompanied by a decrease in RTs (Figure 3A–E). LB intervention caused abnormally increased gamma oscillations and reduced beta oscillations in the M1 in the PD + LB group relative to those in the PD + saline group (beta, *p* < 0.0001; gamma, *p* = 0.0006; Figure 3F,H–J). In the DLS, gamma oscillation power was greater in the PD + LB group than in the PD + saline group (*p* = 0.0022); no difference in beta activity was detected (beta, *p* = 0.0815; Figure 3G,K–M).

### 3.5 Abnormal Functional Connectivity in the CBTC Circuit in the LID State

**FIGURE 4:**
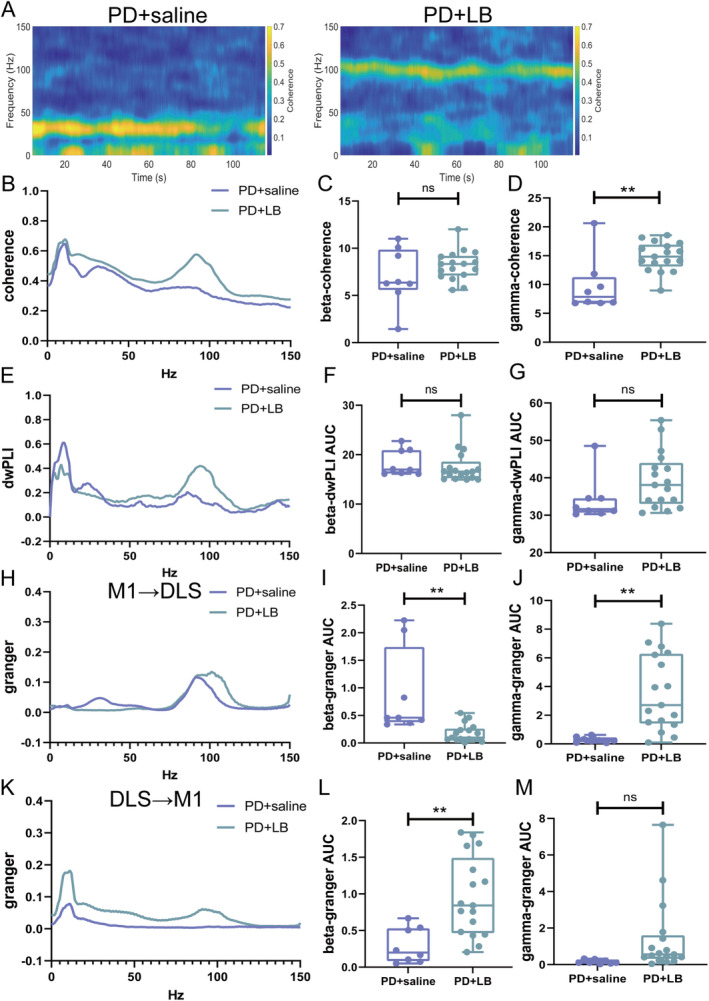
Functional connectivity in the CBTC circuit in the LID state. (A) Representative spectrum plots of coherence between the M1 and DLS from the PD + saline and PD + LB groups. (B) M1–DLS coherence. (C and D) Summaries of coherence in the beta and gamma bands. (E) M1–DLS dwPLIs. (F, G) Summaries of beta‐ and gamma‐band dwPLIs. (H–J) M1 → DLS causality. (K–M) DLS → M1 causality. Data are means ± SEMs. **p < 0.01, unpaired *t*‐test (PD + saline, *N* = 8; PD + LB, *N* = 17). DLS, dorsolateral striatum; dwPLI, directed weighted phase lag index; LB, levodopa plus benserazide; LFP, local field potential; M1, motor cortex; PD, Parkinson's disease; PSD, power spectrum densities; SEM, standard errors of mean.

The functional connectivity in the CBTC circuit was aberrant in the LID state. Gamma‐band coherence was greater in the PD + LB group than in the PD + saline group (*p* = 0.0019; Figure 4A,B,D); no difference in beta‐band coherence (*p* = 0.2126; Figure 4C) or beta‐ or gamma‐band dwPLIs (beta, *p* = 0.5523; gamma, *p* = 0.1935; Figure 4E–G) was detected. In the M1 → DLS direction, beta causality was greater in the PD + saline group (*p* = 0.0013; Figure 4H,I), and gamma causality was greater in the PD + LB group (*p* = 0.002; Figure 4J). In the DLS → M1 direction, beta causalities were increased in the PD + LB group (beta, *p* = 0.0031; Figure 4K,L), whereas gamma oscillation did not differ between the groups (*p* = 0.0954; Figure 4M).

### 3.6 Effect of Tetrabenazine on the Motor Performances and Neural Activities of Rats With LID

**FIGURE 5:**
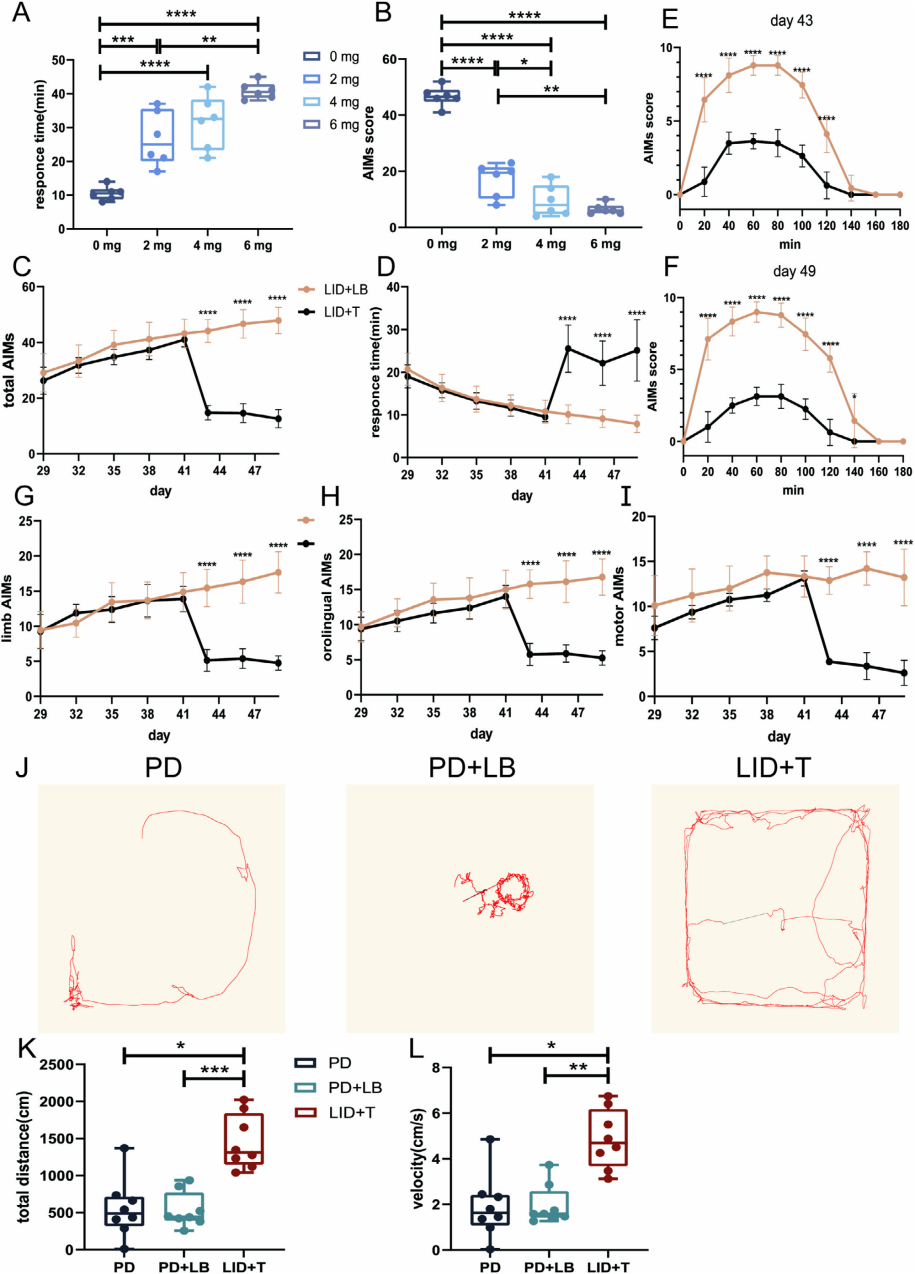
Effects of tetrabenazine on the motor performance of rats with LID. (A and B) RTs and total AIMS scores after LB administration and tetrabenazine intervention. (C, E, and F) Total AIMS scores after LB priming, overall and on Days 43 and 49. (D) RTs after LB administration. (G–I) AIMS subscale scores after LB priming in the LID + LB and LID + T groups. (J) Representative open‐field test motion trails. (K and L) Intergroup comparisons of total distance traveled and velocity of LID + T group rats in PD, LID, and post‐tetrabenazine intervention states. Data are means ± SEMs. *p < 0.05, **p < 0.01, ***p < 0.001, and ****p < 0001, unpaired *t*‐test and RM one‐way ANOVA test followed by Tukey's multiple comparison test (0, 2, 4, and 6 mg, *N* = 5 for each group; LID + LB, *N* = 9; LID + T, *N* = 8; PD, *N* = 8; PD + LB, *N* = 8; LID + T, *N* = 8). AIMs, Abnormal Involuntary Movement Scale; ANOVA, analysis of variance; DLS, dorsolateral striatum; LB, levodopa plus benserazide; LID, levodopa‐induced dyskinesia; M1, motor cortex; RM, repeated measure; RTs, response times; SEM, standard errors of mean; T, tetrabenazine.

After 14 consecutive days of LB administration, a stable LID model was established. The tetrabenazine intervention ameliorated dyskinetic symptoms, reducing Day 15 AIM scores (one‐way ANOVA, *F*(3, 20) = 96.82, *p* < 0.0001; Tukey's multiple comparisons test: 0 vs. 2, 4, and 6 mg, all *p* < 0.0001; 2 vs. 4 mg, *p* = 0.0459; 2 vs. 6 mg, *p* = 0.0038; Figure 5B) and increasing RTs (one‐way ANOVA, *F*(3, 20) = 28.14, *p* < 0.0001; Tukey's multiple comparisons test: 0 vs. 2 mg, *p* = 0.0006; 0 vs. 4 and 6 mg, both *p* < 0.0001; 2 vs. 6 mg, *p* = 0.0024; Figure 5A). The AIM scores were much lower and RTs were much longer with 6 and 4 mg of tetrabenazine than with 2 mg of tetrabenazine, with no difference between the 4 and 6 mg of tetrabenazine. Thus, 4 mg/kg tetrabenazine was used in subsequent behavioral and electrophysiological analyses. On Days 43, 46, and 49, the LID + T group had reduced AIM scores and prolonged RTs (AIM scores, all *p* < 0.0001; RTs, all *p* < 0.0001; Figure 5C,D). Moreover, the AIM scores decreased at each 20‐min interval from 20 to 140 min on Days 43 and 49 (unpaired *t*‐test, min, all *p* < 0.0001; min, all *p* < 0.0001; 140 min, *p* = 0.0467; Figure 5E,F). The AIM subscale scores showed the same decreasing trend on Days 43, 46, and 49 (orolingual, all *p* < 0.0001; forelimb, all *p* < 0.0001; motor, all *p* < 0.0001; Figure 5G–I).

Additionally, the locomotor activities of LID + T group rats on Day 14 (PD state, PD), Day 42 (LID state, PD + LB), and Day 48 (post‐tetrabenazine intervention state, LID + T) were evaluated via the OFT. LID + T group rats exhibited increased locomotor activity with increased movement distance and velocity relative to PD + LB and LID + LB group rats on Day 48 (repeated‐measures one‐way ANOVA, total distance: *F*_2,22_ = 15.36, *p* = 0.0011; Tukey's multiple comparisons test: PD vs. LID + T, *p* = 0.0137; PD + LB vs. LID + T, *p* = 0.0007; velocity: *F*_2,22_ = 12.59, *p* = 0.0024; Tukey's multiple comparisons test: PD vs. LID + T, *p* = 0.0226; PD + LB vs. LID + T, *p* = 0.0022; Figure 5J–L).

**FIGURE 6:**
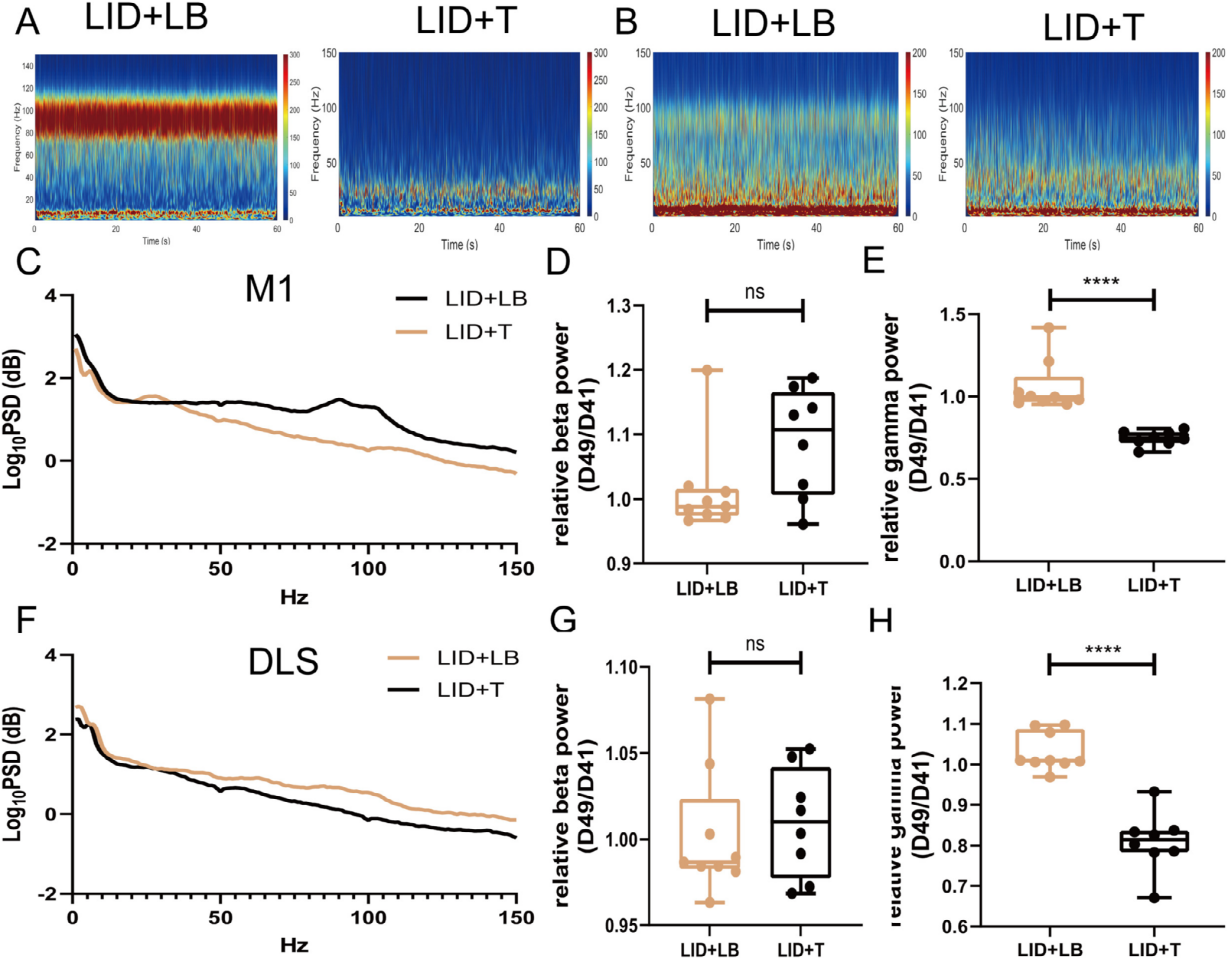
Effects of tetrabenazine on the neural activities of rats with LID. (A and B) Representative M1 and DLS PSD spectrum plots from the LID + LB and LID + T groups. (C and F) PSDs of LFPs in the M1 and DLS. (D, E, G, and H) Summaries of normalized beta‐ and gamma‐band oscillation in the M1 and DLS. Data are means ± SEMs. ****p < 0001, unpaired *t*‐test (LID + LB, *N* = 9; LID + T, *N* = 8). DLS, dorsolateral striatum; LB, levodopa plus benserazide; LFP, local field potential; LID, levodopa‐induced dyskinesia; M1, motor cortex; PSD, power spectrum densities; SEM, standard errors of mean; T, tetrabenazine.

**FIGURE 7:**
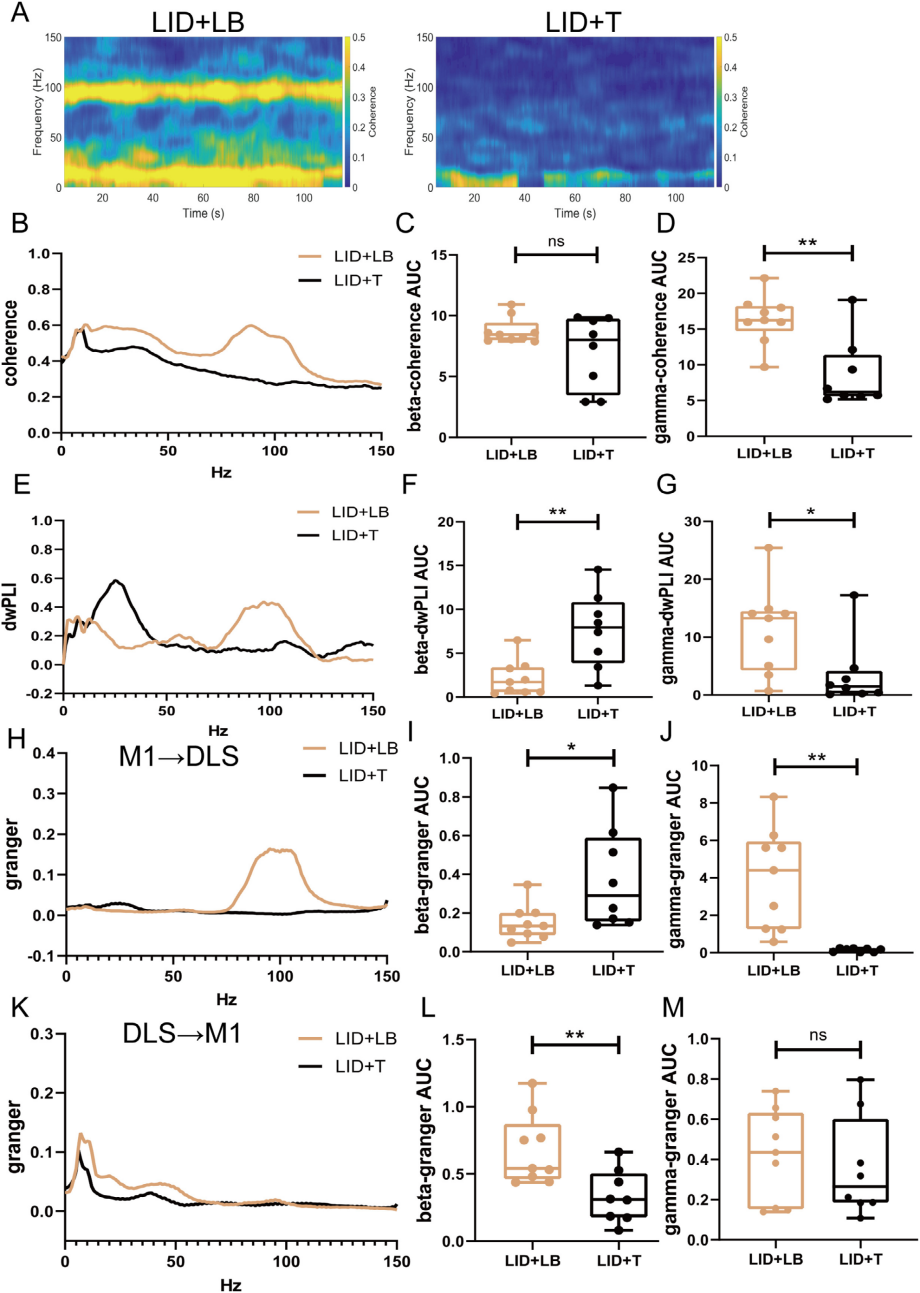
Neural functional connectivity alterations in the CBTC circuit after tetrabenazine administration. (A) Representative spectrum plots of coherence between the M1 and DLS. (B) M1–DLS coherence. (C and D) Summaries of beta‐ and gamma‐band coherence. (E) M1–DLS dwPLIs. (F and G) Summaries of beta‐ and gamma‐band dwPLIs. (H–J) M1 → DLS causality. (K–M) DLS → M1 causality. Data are means ± SEMs. *p < 0.05 and **p < 0.01, unpaired *t*‐test (LID + LB, *N* = 9; LID + T, *N* = 8). CBTC, cortico‐basal ganglia‐thalamo‐cortical; DLS, dorsolateral striatum; dwPLI, directed weighted phase lag index; LB, levodopa plus benserazide; M1, motor cortex; SEM, standard errors of mean; T, tetrabenazine.

On Day 49, gamma oscillation power in the M1 and DLS was lower in the LID + T group than in the LID + LB group (M1, *p* < 0.0001; DLS, *p* < 0.0001; Figure 6A–C,E,F,H); no difference in beta‐band power was observed (M1: beta, *p* = 0.0671; DLS: beta, *p* = 0.6541; Figure 6D,G). Relative to the LID + LB group, the LID + T group presented an increased beta‐band dwPLI (*p* = 0.0036; Figure 6E,F) and reduced gamma‐band coherence and dwPLI (coherence, *p* = 0.0017; dwPLI, *p* = 0.0335; Figure 7A,B,D,G); no difference in beta‐band coherence (*p* = 0.122; Figure 7C) was observed. Compared with the LID + LB group, the LID + T group presented greater beta causality (*p* = 0.0258; Figure 7H,I) and less gamma causality (*p* = 0.0011; Figure 7J) in the M1 → DLS direction. The LID + LB group showed greater beta causality in the DLS → M1 direction (*p* = 0.0085; Figure 7K,L). No difference in gamma oscillation in the DLS → M1 direction was observed (gamma, *p* = 0.6035; Figure 7M).

### 3.7 Comparison of the Effects of Tetrabenazine and Eltoprazine on the Neural Activities of Rats With LID at the Electrophysiology Level

In a study published by our team last year, we demonstrated that eltoprazine, a 5‐HT1A/B autoreceptor agonist, also reduces gamma oscillations in the M1 → DLS direction, leading to a reduction in dyskinesia symptoms [43]. On the basis of these findings, we compared the efficacy of eltoprazine and tetrabenazine in alleviating LID symptoms at the electrophysiological level (Figure S2A,E). We measured changes in beta‐ and gamma‐band power on Days 49 and 43 after intervention to evaluate therapeutic effects. In the M1 region, eltoprazine had greater effects on both beta and gamma oscillation power than did tetrabenazine (beta, *p* < 0.0001; gamma, *p* < 0.01; Figure S2B–D). Analysis of PSDs in the DLS revealed a distinct pattern, with eltoprazine more effectively decreasing beta oscillation, whereas tetrabenazine had a stronger effect on reducing gamma oscillation power (beta, *p* < 0.0001; gamma, *p* < 0.001; Figure S2E–G).

## 4 Discussion

To better understand the pathological mechanisms underlying PD and LID at the electrophysiological level, we investigated neural electrophysiological alterations in PD and LID model rats in this study. We also explored the electrophysiological changes occurring after tetrabenazine intervention in rats with LID to clarify the mechanism underlying the therapeutic effects of this drug.

In our study, we observed that beta power was significantly increased in the M1 and DLS of PD model rats. However, in LID model rats, beta power was reduced, whereas gamma oscillation power was notably increased. Interestingly, there was no significant difference in gamma oscillation power between the PD and LID model rats.

Research into the neural electrophysiology of LID has focused predominantly on gamma oscillations, which are often considered potential biomarkers for this condition. Recent studies have also explored gamma‐ and theta‐band oscillations in animal models of LID [14, 33]. However, our analyses did not reveal differences in theta‐band power between the PD and LID states, suggesting that gamma rhythms play a more critical role in the LID state. Our findings underscore the importance of gamma rhythms in LID and suggest that targeting abnormal gamma oscillations could offer new avenues for noninvasive treatments. For example, gamma oscillations might be leveraged in noninvasive stimulation techniques or closed‐loop DBS to improve the efficacy of LID therapy. The development of methods to modulate these gamma rhythms could lead to more practical, cost‐effective therapies for central nervous system (CNS) diseases and accelerate their clinical implementation. We observed abnormal functional connectivity in the CBTC circuit in this study; the administration of 6‐OHDA significantly increased beta‐band coherence and the dwPLI, indicating enhanced synchronization and functional connectivity between brain regions in the beta frequency range. In contrast, the injection of LB primarily elevated gamma‐band coherence, suggesting altered communication within brain networks at higher frequencies. These results highlight distinct neural network changes associated with different pathological models, potentially contributing to our understanding of disease mechanisms in neurodegeneration. Thus, neural network alterations in these pathological states are distinct. Our findings contribute to the study of M1‐related functional connectivity in the pathophysiological processes of PD and its complications, providing a foundation for evaluating the regulation of subcortical neural network connectivity in subjects with LID. In recent years, analytical techniques that combine functional and structural connectivity analyses have been used to characterize the profiles of patients undergoing DBS, and the abnormal changes in functional connectivity in the LID state observed in this study may provide a basis for the selection of patients that would benefit from DBS and prediction of the clinical efficacy of this treatment.

This study revealed that beta causality in the M1 → DLS direction is abnormally strengthened in the PD state and that chronic l‐DOPA administration suppresses this effect, increases gamma causality in the M1 → DLS direction, and increases beta causality in the DLS → M1 direction. The extent to which information flow is altered in local CBTC regions in the PD and LID states and the potential contributions of this alteration to clinical manifestations have not been fully elucidated; thus, the investigation of electrophysiological connectivity between these nodes is relevant [44]. A multilevel analysis revealed different patterns and mediations of causal connectivity in the ventral intermediate nucleus among PD subtypes [45]. Consistent with our findings, a previous study revealed increased beta causality in the PD state and increased theta causality in the dyskinetic state in the DLS → SNr direction [40]. Our results confirmed the directionality of the information transfer direction between the M1 and DLS, representing the source and destination of CBTC motor circuit signals in diverse disease states. We further clarified the effects of LB injection on information flow and directionality, including the strengthening of beta causality in the DLS → M1 direction. STN DBS has been shown to shift hierarchical organization and alter information flow in prefrontal networks, which suggests that the underlying mechanism is neurophysiological [46]. Our exploration of message transfer in the CBTC circuit enabled the characterization of various pathological states at the neural electrophysiological level, providing a new target for DBS treatment and detection of underlying mechanisms.

We demonstrated that tetrabenazine, which targets VMAT‐2, affected dyskinetic symptoms and aberrant neural activities in the LID state and thus could be used to treat LID. The tetrabenazine intervention reduced AIM scores and increased RTs in a dose‐dependent manner, suppressed abnormally exaggerated gamma oscillation without altering theta or beta activity, affected the direction of information flow with increased beta causality, reduced the strengthened functional connectivity of synchronized gamma rhythms and increased that in the beta band in the CBTC circuit, and suppressed gamma causality in the M1 → DLS direction and beta causality in the DLS → M1 direction. Recent research has demonstrated that the mechanism of PD involves a reduction in cytoplasmic catecholamine vesicular sequestration related to VMAT and aldehyde dehydrogenase activity in residual neurons with specific functional abnormalities [47]. VMAT‐2 inhibitors, which impede the exaggerated dopaminergic activity resulting from prolonged antipsychotic use by disturbing dopamine uptake and storage, have shown potential efficacy for the treatment of tardive dyskinesia [35]. Our research confirmed the effect of tetrabenazine on subjects with LID. Previous studies have demonstrated that high gamma power in the M1 is correlated with AIMs [12]. Therefore, we hypothesize that tetrabenazine alleviates LID symptoms by modulating this aberrant gamma activity. For the first time, we clarified the mechanism underlying the effects of tetrabenazine at the electrophysiological level. We also examined the effects of tetrabenazine on locomotor activity to prevent the deterioration of PD while restraining LID. According to our observations and data analysis, LB injection induced only pathological involuntary movements, with no effect on locomotion, in contrast to the 6‐OHDA injection. Nevertheless, tetrabenazine intervention ameliorated dyskinetic symptoms and restored locomotor activity, which provided evidence that tetrabenazine exerts antidyskinetic effects without reducing the antiparkinsonian efficacy of l‐DOPA. Our results provide evidence supporting the application of tetrabenazine in the further clinical treatment of LID.

Additionally, we compared the results of this study with those of our previous work. Both tetrabenazine and eltoprazine effectively alleviate dyskinesia symptoms by reducing gamma power and rebalancing functional connectivity and information flow, highlighting their potential clinical relevance for LID management. However, these medications may induce distinct electrophysiological changes. Specifically, our analysis revealed that eltoprazine primarily decreased beta‐ and gamma‐band power in the M1, whereas tetrabenazine notably reduced gamma‐band power in the DLS. The complex mechanisms underlying LID development due to chronic L‐DOPA therapy are still not fully understood. Research indicates that both dopaminergic and nondopaminergic receptors play roles in LID pathogenesis, with changes observed from local synaptic activity to mesoscale hypersynchronization and whole‐brain macroscale connectivity. Although tetrabenazine and eltoprazine modulate dopamine levels through different pathways, they may ultimately affect overlapping neural circuits to produce therapeutic effects. Furthermore, the differing effects on 5‐HT1A/B and VMAT‐2 receptors contribute to the varying electrophysiological alterations induced by these drugs. Importantly, our comparison with previous studies is preliminary and may be influenced by confounding factors due to potential variations in experimental settings, despite the use of a consistent protocol and framework. Consequently, further research specifically targeting different dopaminergic and nondopaminergic receptors is essential to identify the most effective therapeutic agents for clinical LID treatment.

However, the findings of this study have several limitations. For decades, extensive research has been dedicated to elucidating the mechanisms underlying neural oscillations. Recent studies suggest that periodic bursts of activity in single neurons may play a crucial role in encoding the pathophysiological β‐band oscillations (13–33 Hz) observed in LFPs within the STN in PD. These beta oscillations are being increasingly recognized as key markers of motor dysfunction in PD, reflecting disrupted neural network dynamics and providing insights into disease progression and potential therapeutic targets. By integrating advances in neurophysiological recording techniques and computational modeling, researchers are now able to explore the complex interplay between neuronal firing patterns and beta oscillations, enhancing our understanding of the underlying mechanisms and informing the development of targeted interventions for managing parkinsonian symptoms [48]. However, our study focused primarily on large‐scale neural oscillations and did not directly assess single‐neuron activity. This is a limitation, as examining both levels of neural activity could offer more comprehensive insights into how pathological beta oscillations are generated and maintained. Future studies should aim to incorporate both single‐neuron burst analysis and LFP recordings to provide a more complete picture of the neural mechanisms underlying beta oscillations and their relationship with motor symptoms. In addition, a pathological striatal circuit state resulting from dopamine depletion, in which distinct striatal neuron subtypes coordinate selectively with specific oscillations during locomotion, has been characterized [49]. However, little is known about how specific cell types in brain regions involved in the CBTC circuit support the generation, propagation, and interaction of oscillatory dynamics throughout CBTC circuits or how specific oscillatory dynamics are related to motor function. Optogenetics and more advanced technologies are needed for further exploration and precise regulation. Although previous studies have demonstrated the treatment effect of tetrabenazine on LID animal models and patients, multicenter, double‐blind, large cohort clinical studies are needed for its application in LID therapy in the future. In addition, we confirmed the potential electrophysiological mechanism of tetrabenazine, but its molecular mechanisms and effects on synaptic plasticity are still unclear.

In summary, we characterized the distinct neural electrophysiological patterns occurring in the PD and LID states and confirmed that tetrabenazine exerts its antidyskinetic effects via the normalization of aberrant neural activity. We demonstrated that 6‐OHDA injection increased beta oscillation and that l‐DOPA administration generated exaggerated gamma oscillation in the M1 and DLS. Alterations in functional connectivity, represented by coherence and the dwPLI, were also observed in the parkinsonian and dyskinetic states. In addition, information flow in the corticostriatal circuit differed between these states. Finally, we confirmed that tetrabenazine abrogates aberrant gamma oscillation to improve LID symptoms, providing compelling evidence for its future clinical application in LID therapy.

## Author Contributions

Y.B. and W.Z. contributed to the conception and design of the study. Y.B., P.W., M.L., Z.W., S.L., and Y.Y. contributed to the acquisition and analysis of the data. Y.B. and P.W. contributed to drafting the text and preparing the figures.

## Conflicts of Interest

The authors declare no conflicts of interest. Some of the figures were created with Biorender.com.

## Supporting information


Figures S1 and S2.

## Data Availability

The data that support the findings of this study are available from the corresponding author upon reasonable request.
