# Supplementary material for: Electrophysiological Alterations in the Progression of Parkinson's Disease and the Therapeutic Effect of Tetrabenazine on Rats With Levodopa‐Induced Dyskinesia
Source: CNS Neurosci Ther. 2025 Feb 6;31(2):e70250. doi: 10.1111/cns.70250 (PMC11799927; doi:10.1111/cns.70250)
Supplement: Supplementary file 1 — Figures S1 and S2. [file CNS-31-e70250-s001.docx]

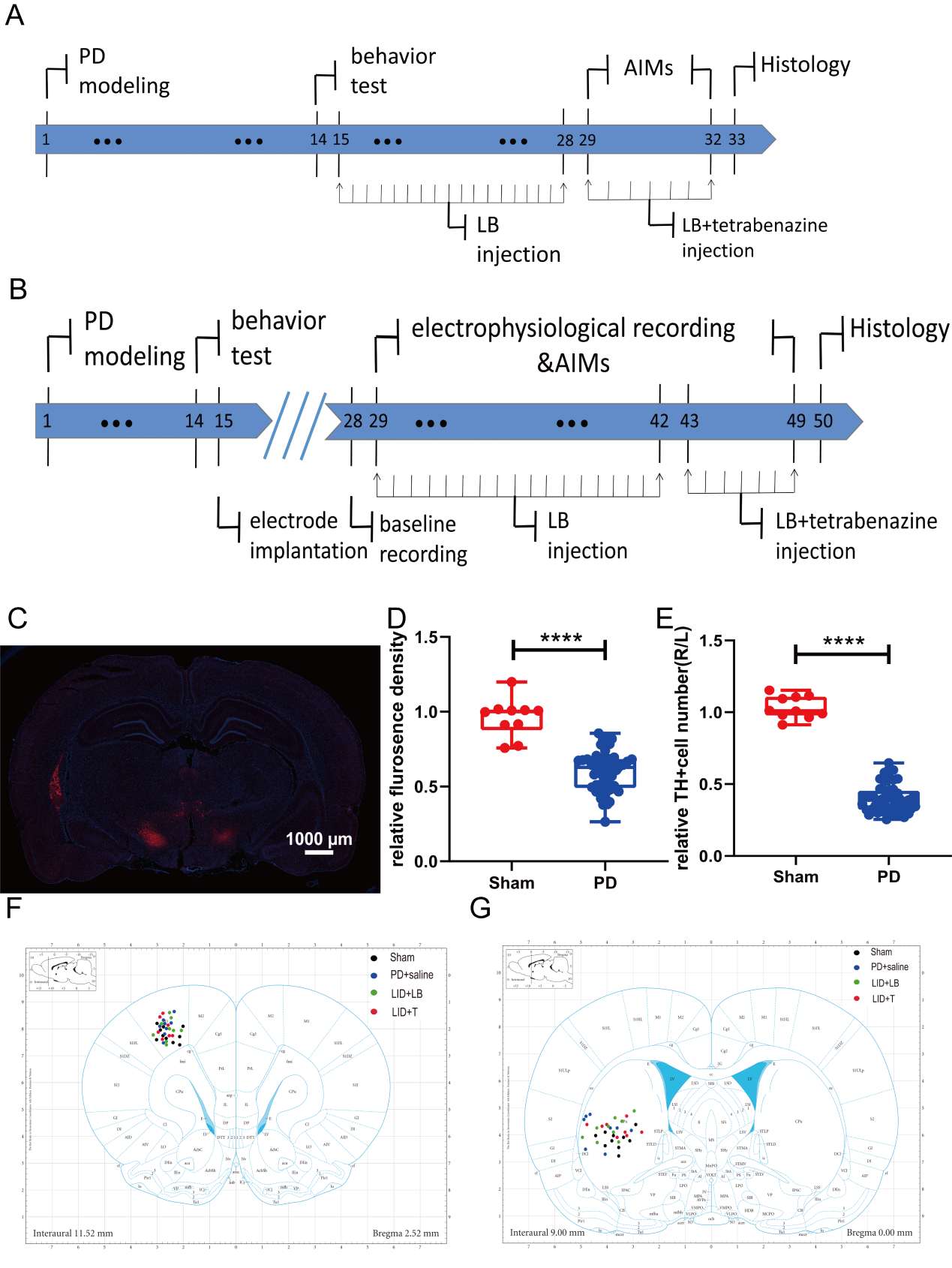


Supplementary Figure 1.

1. Schematic diagram of the experiment 1 procedure. B. Schematic diagram of the experiment 2 procedure. C. Representative TH *immunofluorescence* labeling of DA neurons in the SNc in rats with PD. D. Relative TH immunofluorescence density in the SNc. E. Relative numbers of TH+ cells (red, representing the TH-immunopositivity neurons, which indicating dopaminergic neurons, the corresponding fluorescent indicator is Alexa Fluro 594 donkey anti-rabbit antiserum) in the SNc. F, G. Electrode implantation sites in the M1 and DLS. Data are means ± SEMs. *****p* < 0001, Kolmogorov–Smirnov and unpaired *t* tests.

DLS, dorsolateral striatum; M1, motor cortex; PD: Parkinson’s Disease; SNc, substantia nigra pars compacta; SEM, standard errors; TH, tyrosine hydroxylase.


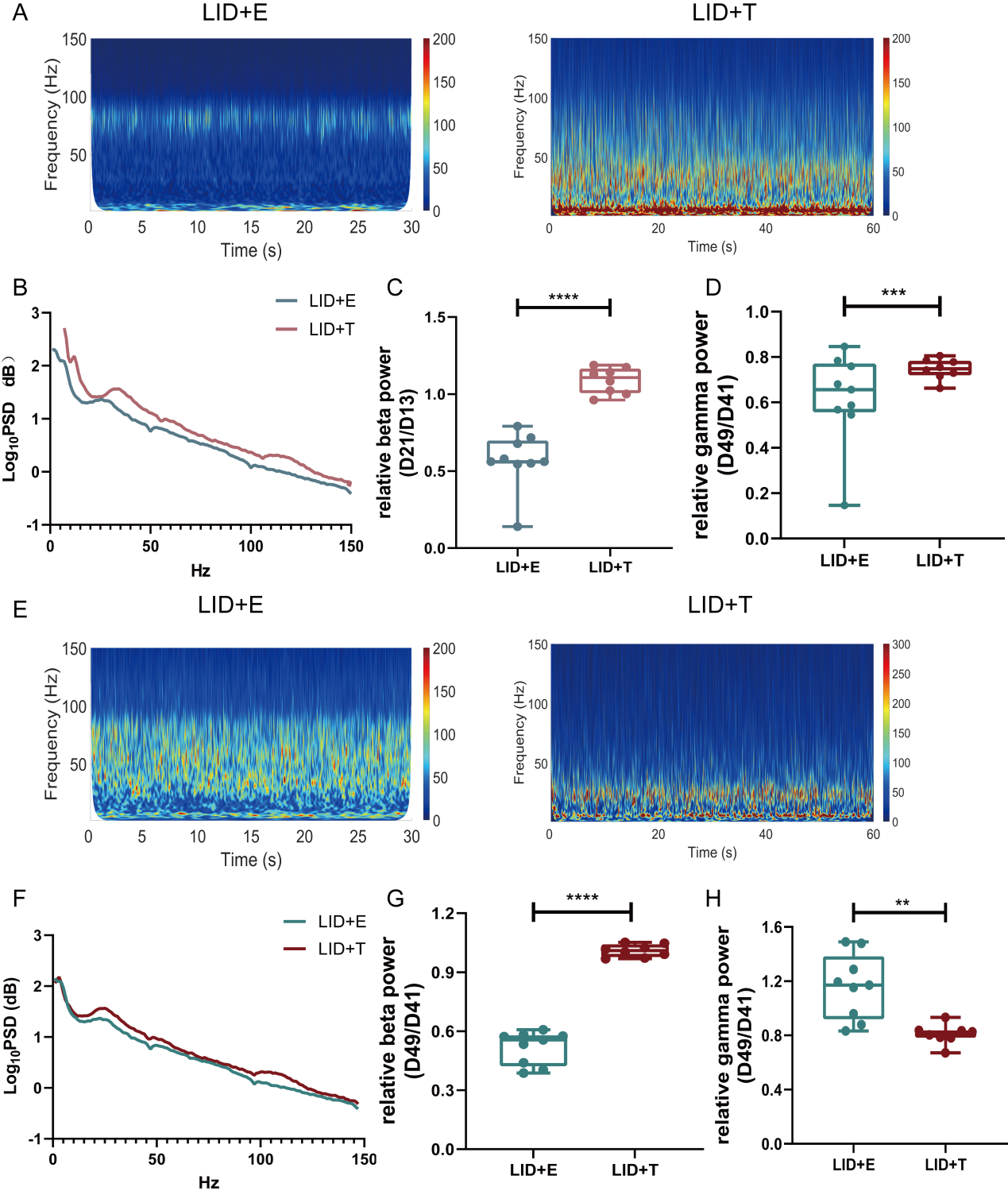


Supplementary Figure 2.

A, E. Representative M1 and DLS PSD spectrum plots from the LID+E and LID+T groups. B, F. PSDs of LFPs in the M1 and DLS. D, E, G, H. Summaries of normalized beta- and gamma-band oscillation power in the M1 and DLS. Data are means ± SEMs. **p < 0.01, ***p < 0.001, ****p < 0001, unpaired t test (LID+E, N = 9; LID+T , N = 8).
